# Revisiting organic charge-transfer cocrystals for wide-range tunable, ambient phosphorescence†

**DOI:** 10.1039/d3sc04001a

**Published:** 2023-10-31

**Authors:** Anju Ajayan Kongasseri, Shagufi Naz Ansari, Swadhin Garain, Sopan M. Wagalgave, Subi J. George

**Affiliations:** a New Chemistry Unit and School of Advanced Materials, Jawaharlal Nehru Centre for Advanced Scientific Research (JNCASR) Jakkur Bangalore 560064 India george@jncasr.ac.in

## Abstract

Simple and efficient designs that enable a wide range of phosphorescence emission in organic materials have ignited scientific interest across diverse fields. One particularly promising approach is the cocrystallization strategy, where organic cocrystals are ingeniously formed through relatively weaker and dynamic non-covalent interactions. In our present study, we push the boundaries further by extending this cocrystal strategy to incorporate donor–acceptor components, stabilized by various halogen bonding interactions. This non-covalent complexation triggers ambient, charge-transfer phosphorescence (^3^CT), which can be precisely tuned across a broad spectrum by a modular selection of components with distinct electronic characteristics. At the core of our investigation lies the electron-deficient phosphor, pyromellitic diimide, which, upon complexation with different donors based on their electron-donating strength, manifests a striking array of phosphorescence emission from CT triplet states, spanning from green to yellow to reddish orange accompanied by noteworthy quantum yields. Through a systematic exploration of the electronic properties using spectroscopic studies and molecular organization through single-crystal X-ray diffraction, we decisively establish the molecular origin of the observed phosphorescence. Notably, our work presents, for the first time, an elegant demonstration of tunable ^3^CT phosphorescence emission in intermolecular donor–acceptor systems, highlighting their immense significance in the quest for efficient organic phosphors.

## Abstract 2

A wide-range tunable triplet charge-transfer (^3^CT) phosphorescence emission is achieved by an intermolecular through-space charge-transfer cocrystallization approach using pyromellitic diimide as the acceptor and different donors.

## Graphical abstract

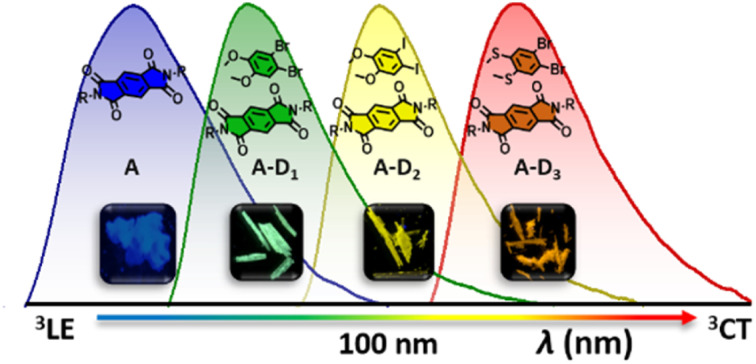

## Introduction

Realizing ambient, room temperature phosphorescence is one of the most actively pursued research currently, because of its huge potential in the realm of solution processable sustainable energy sources for various applications like solid-state lighting, displays, information encryption and so on.^1^ However, efficient triplet harvesting of organic molecules to achieve ambient phosphorescence requires careful and clever molecular design due to their generally weak spin–orbit coupling (SOC), and quenching of triplet excited states *via* various non-radiative decay processes and oxygen. In the recent past, this field has witnessed an upsurge of many molecular strategies and synthetic modifications to advance the phosphorescence efficiencies by enhancing the inter-system crossing (ISC) rate and minimizing the deactivating mechanisms of triplet states.^2–4^

Recently, cocrystallization has been shown as an efficient strategy to improve the efficacy of organic phosphors. Initiated by the seminal work of Kim and co-workers, where they have demonstrated a ‘directed’ heavy-atom effect to boost the performance of organic phosphors *via* cocrystallization to minimize π–π stacking induced luminescence quenching (dilution), this strategy has been revolutionized by different groups.^2*a*^ In this organic cocrystal strategy, non-covalent interactions such as hydrogen bonding, ionic interactions and π–π stacking between the molecular components provides a rigid framework by bringing them in close proximity and thus preventing vibrational dissipation, thereby complementing the offsets by enhancing the phosphorescence efficiency. Among various interactions, the use of halogen bonding has an advantage in the design of cocrystal phosphors by enhancing the SOC of the system *via* the heavy atom effect from the complementary component.^5^ However, realizing wide range, tunable phosphorescence is considered as one of the next grand challenges in this field and it is often achieved by structurally perturbing the organic phosphor. An alternative design of introducing lower energy intramolecular charge-transfer (ICT) states *via* clever donor–acceptor molecular designs is shown to get tunable triplet state emission, new triplet harvesting pathways such as thermally activated delayed fluorescence (TADF) and also accelerating the process of ISC in accordance with El-Sayed's rule.^6^ However, the cocrystallization strategy often helps in enhancing the RTP properties by amplifying the phosphorescence emission from the locally excited triplet state (^3^LE) of phosphors.^5^ In this context, we envisage that if we can extend the cocrystallization strategy to electronically complementary donor and acceptor components with heavy atoms, then the scope of this strategy could be extended to explore the through-space intermolecular charge-transfer (CT) states. Furthermore, this design would help in biasing the triplet harvesting pathways and also to tune the emission *via* modulating the electronic characteristics of one of the components, thereby providing a modular approach to tune emission from the organic phosphors.

**Fig. 1:**
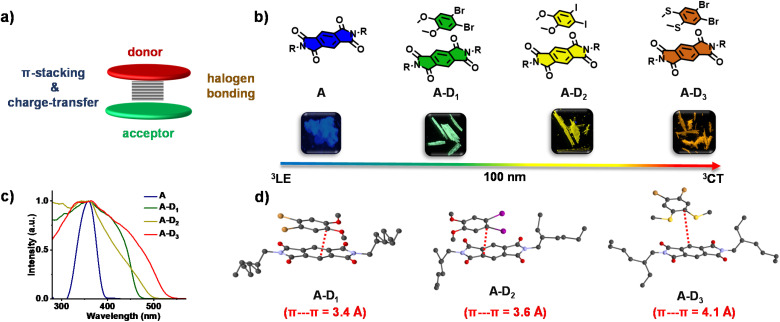
(a) Schematic illustrating the design of non-covalent donor–acceptor cocrystals. (b) Molecular structures of pyromellitic diimide (A) and corresponding donor–acceptor cocrystals (A–D_1_, A–D_2_ and A–D_3_) with the photographs of their cocrystals (under a 365 nm UV lamp) and emission of A and the corresponding cocrystals (A–D_1_, A–D_2_ and A–D_3_) showed a clear red-shift of 100 nm from cyan to green to yellow to reddish orange. (c) Excitation spectra of A (*λ*_monitored_ = 500 nm) and corresponding cocrystals (A–D_1_, A–D_2_ and A–D_3_; *λ*_monitored_ = 515 nm, 555 nm and 595 nm, respectively), which showed a red-shifted band compared to A, signifying the CT interaction. (d) Single-crystal X-ray diffraction data showing the π–π interactions and corresponding distances (shown in red dashed lines) in cocrystals.

We explored this possibility using a pyromellitic diimide acceptor, an efficient phosphor reported by our group, which is shown to have excellent triplet quantum yields unlike other arylene diimide analogues.^5*a*,7^ We have recently exploited the ability of arylene diimides to form CT complexes with various donor molecules,^8^ to bias the excited state manifold of pyromellitic diimides.^5*a*^ Motivated by these results, to extract phosphorescence emission from the through-space CT states and to tune the emission *via* a modular non-covalent approach, herein we report cocrystallization of the acceptor with a thoughtful choice of heavy atom substituted electron rich molecules. While the crystallization of electronically complementary donor–acceptor systems occurs *via* through space CT states, the heavy atoms present in the donor and consequent halogen bonding ensure the sensitization of ^3^CT phosphorescence (Fig. 1a). Through this approach, herein we show unprecedented wide range tunable CT phosphorescence from ^3^CT states over an emission range of 100 nm by using a series of donor molecules. Although various phosphor designs are reported, the current non-covalent donor–acceptor cocrystal approach, being low-cost, feasible and devoid of any cumbersome synthesis with multiple steps, forges the strategy ideal for synthesizing a diverse library of molecules with tunable emission. Here, we have chosen a series of heavy-atom substituted donors with varying electron donating strength such as 1,2-dibromo-4,5-dimethoxy benzene (D_1_), 1,2-diiodo-4,5-dimethoxy benzene (D_2_) and (4,5-dibromo-1,2-phenylene)bis(methylsulfane) (D_3_) (Fig. 1b). An unsubstituted pyromellitic diimide phosphor, A, appended with branched 2-ethylhexyl side chains was chosen as the acceptor component so as to enhance the solubility and to facilitate incorporation of donor components.

## Results and discussion

Initially, we have synthesized the donor and acceptor molecules and characterized them using NMR and mass spectrometry (Schemes S_1_–S_4_, Fig. S10–S18†). With an objective to achieve tunable red-shifted emission, we have grown 1 : 1 cocrystals of A with D_1_, D_2_ and D_3_ (A–D_1_, A–D_2_ and A–D_3_, respectively). All the cocrystals were luminescent with shiny green, yellow and reddish orange-coloured emissions, respectively (Fig. 1b). Single-crystal X-ray diffraction (SCXRD) studies suggested the formation of cocrystals with donor–acceptor stacked organization as evidenced from the π-stacking (Fig. 1d). The excitation spectra of the cocrystals showed intense, red-shifted bands (*λ*_monitored_ = 515, 555 and 595 nm, respectively for A–D_1_, A–D_2_ and A–D_3_) compared to that of the acceptor, which further confirmed the alternate donor–acceptor organization and CT interactions, as envisioned (Fig. 1c). The well-obvious red-shift observed in the excitation spectra delineated towards a different source of origin other than acceptor LE states, which could be possibly from CT states (*vide infra*, Fig. 1c). Furthermore, the highly red-shifted absorption band in the UV-Vis absorption spectra of all cocrystals compared to the acceptor alone confirmed the formation of a ground-state charge-transfer complex in all the cocrystals (Fig. S1†).

**Fig. 2:**
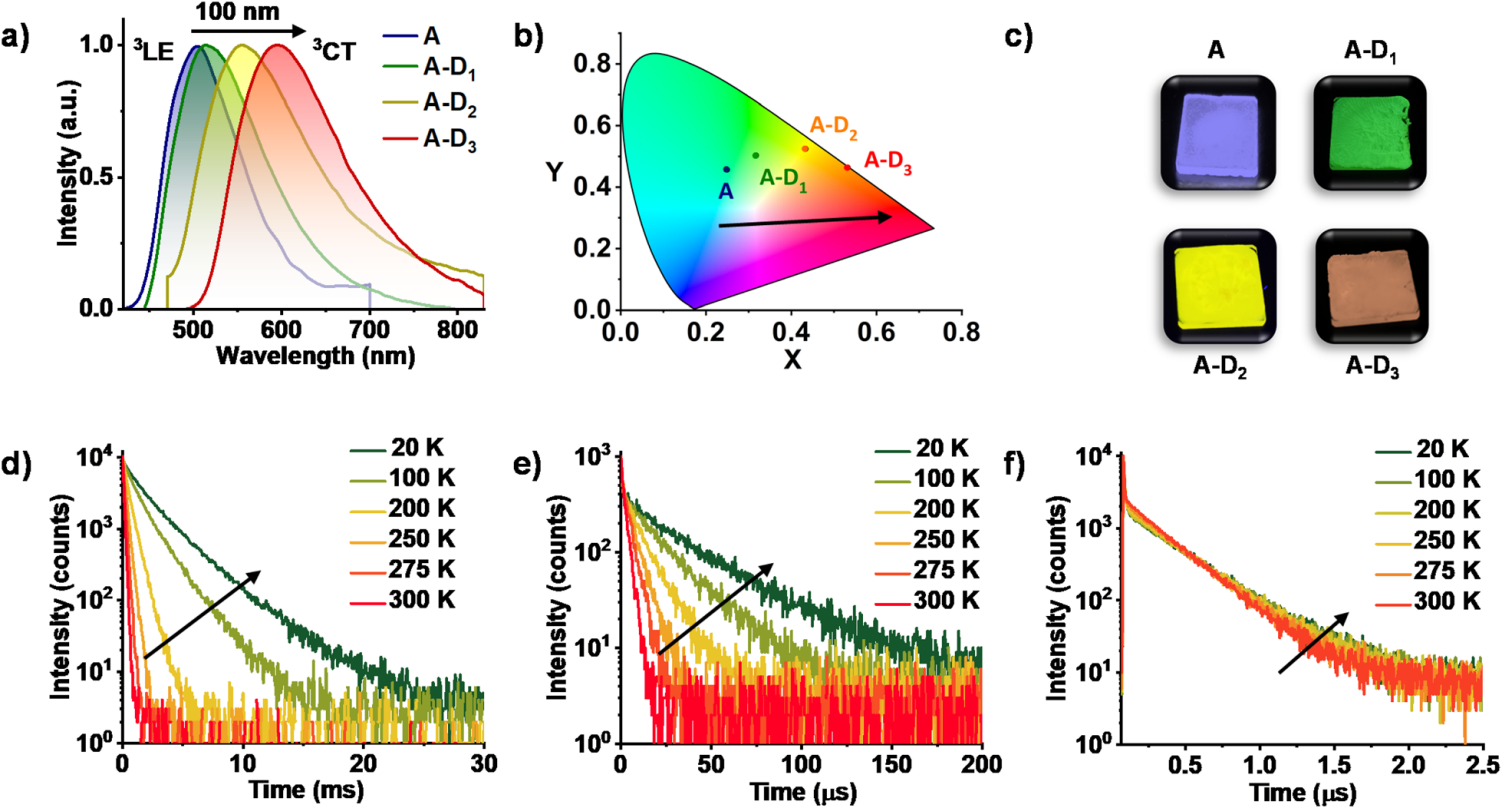
(a) Steady-state emission spectra of A (*λ*_exc._ = 340 nm), A–D_1_ (*λ*_exc._ = 430 nm), A–D_2_ (*λ*_exc._ = 450 nm) and A–D_3_ (*λ*_exc._ = 480 nm), suggesting a gradual red-shift in the emission maxima while progressing from A to A–D_1_, A–D_2_ and A–D_3_. (b) The CIE coordinates of different ^3^CT emissions according to the CIE 1931 chromaticity. (c) Photographs of the films of A and CT complexes taken under a 365 nm UV lamp. Lifetime decay plots of (d) A–D_1_ (*λ*_exc._ = 430 nm and *λ*_collected_ = 515 nm), (e) A–D_2_ (*λ*_exc._ = 405 nm and *λ*_collected_ = 555 nm) and (f) A–D_3_ (*λ*_exc._ = 405 nm and *λ*_collected_ = 595 nm) cocrystals at varying temperatures suggesting the phosphorescence nature.

We have performed detailed spectroscopic studies to understand the modulation of triplet emission of A upon CT complexation. The donor–acceptor cocrystals displayed a diverse emission with maxima spanning from 515 nm to 555 nm to 595 nm, while moving from A–D_1_ to A–D_2_ to A–D_3_ (*λ*_exc._ = 340 nm) (Fig. 2b and S2a†). Furthermore, upon selective excitation of all three cocrystals at the lower energy band (*λ*_exc._ = 430, 450 and 480 nm, respectively), similar emission profiles were observed that confirmed the origin of emission to be the CT states formed during the process of cocrystallization (Fig. 2a). The average lifetimes of A–D_1_, A–D_2_ and A–D_3_ were found to be 95.10 μs, 1.51 μs and 0.22 μs, respectively, hinting towards the presence of a long-lived component in the emission (Fig. S2b, Table S1†). In addition, the photophysical properties of individual donors and acceptors suggested that the origin of emission of the cocrystals is different from the individual triplet states of donors and acceptor (Fig. S3†). In all three cocrystals, the emission intensity and lifetime increased in vacuum when compared to air, which corroborated the triplet contribution to the overall emission (Fig. S4–S6 and Tables S2–S4†).

Subsequently, spectroscopic studies of the three cocrystals at various temperatures were carried out to unambiguously determine the long-lived nature of the emission. All three co crystals exhibited an increased emission intensity and lifetime at low temperatures, which is typical for phosphorescence emission (Fig. 2d–f and S4–S6, Tables S5–S7†). The significantly less lifetime of A–D_2_ and A–D_3_ compared to A–D_1_ can be attributed to the enhanced rate of ISC due to the presence of heavy atoms in accordance with El Sayed's rule. It is noteworthy to mention that, in A–D_3_ cocrystals, a short lifetime component increased with increase of temperature suggesting the contribution of TADF (Fig. 2f). This suggests that the intermolecular donor–acceptor cocrystallization strategy presented here can also be extended to realize TADF by an appropriate choice of donor components. Thus, the studies so far have unambiguously proved the exclusive and tunable CT nature of the phosphorescence from donor–acceptor cocrystals. Further the absolute quantum yields of CT phosphorescence of A–D_1_, A–D_2_, and A–D_3_ in the crystalline state are found to be 45%, 6%, and 14%, respectively, under ambient conditions, suggesting the impressive efficiency of this approach.

**Fig. 3:**
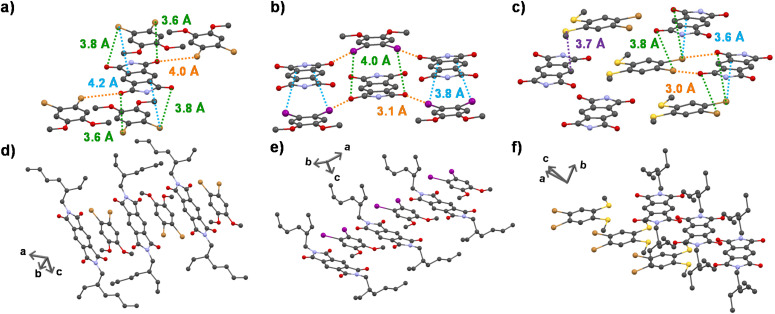
Extended crystal structures of (a) A–D_1_, (b) A–D_2_ and (c) A–D_3_ showing halogen–carbonyl interactions (shown in green dashed lines) in the adjacent planes, halogen–carbonyl interactions (shown in orange dashed lines) in the same plane and halogen–π interactions (shown in blue dashed lines). The sulfur–lone pair interaction is shown in the A–D_3_ cocrystal as a violet dashed line. Extended long-range crystal structures of (d) A–D_1_, (e) A–D_2_ and (f) A–D_3_ cocrystals, respectively.

To obtain a detailed correlation of efficient CT phosphorescence from cocrystals with its molecular organization, we have carefully analyzed the SCXRD data (Fig. 3, S7 and S8†). Both A–D_1_ and A–D_2_ cocrystals are packed in a mixed (alternate) stacking mode with strong π–π overlap (face-to-face). A–D_1_ exhibited a perfect co-facial arrangement with strong π–π interaction with a stacking distance of 3.4 Å (Fig. 1d and S8a†). The excellent co-facial overlap in A–D_1_ augmented an efficient ground state CT complexation, which has resulted in efficient CT phosphorescence. Further analysis showed the presence of various other non-covalent interactions that helped in populating the triplet excited states (Fig. 3a). The halogen–π distance was found to be 4.2 Å in the co-facial arrangement of D and A in the cocrystal. The two C=O⋯Br (halogen–carbonyl) interactions in the same layer (4.0 Å) and four interactions within the adjacent layers (3.6 Å and 3.8 Å) helped to further rigidify the D and A components from vibrational dissipation. Halogen–carbonyl interactions are also known to stabilize the triplet states by an ‘external-heavy atom effect’, thereby increasing the SOC and amplifying the ISC rate, which resulted in an appreciable quantum yield of 45%. Thus, it can be inferred that the multiple halogen–carbonyl interactions present in the A–D_1_ system not only helped in periodically organizing the phosphor, but also in populating the triplet state to enhance its phosphorescence efficiency. It is interesting to mention here that the interdigitated 2-ethylhexyl chains of the acceptors played a significant role in housing the donor *via* non-covalent organization in the cocrystals.

Similar to A–D_1_, A–D_2_ cocrystals are also arranged in a co-facial organization with an interlayer π–π distance of 3.6 Å between alternate D and A components (Fig. 1d and S8b†). Halogen–π (C⋯I = 3.8 Å) and the halogen–carbonyl interactions (C=O⋯I = 4.0 Å (adjacent layers) and C=O⋯I = 3.1 Å (same layer)) assisted in the long-range ordering and enhancing the SOC and ISC rates *via* an external heavy atom effect to result in yellow phosphorescence emission in A–D_2_ (Fig. 3b). We foresee that the relatively less quantum yield of A–D_2_ could be due to the smaller number of halogen bonds (has only four C=O⋯I interactions) compared to A–D_1_ (where six halogen–carbonyl interactions are present), which resulted in a lower population of triplet states in A–D_2_.

In contrast to the above cocrystals, A–D_3_ cocrystals are arranged in a long-range fashion with segregated stacks of A and D_3_ (Fig. 3f). The adjacent columns have D–D–D–D and A–A–A–A stacks with relatively weak π–π overlap. The individual stacks of A and D_3_ are arranged alternately along the *a* axis, perpendicular to the *bc* plane (Fig. 3f). Although weak π–π interaction is observed with a distance of 4.1 Å (Fig. 1d), electron-rich sulfur atoms interact with the electron-deficient π-surface of A within a distance of 3.7 Å (Fig. 3c). Furthermore, two electron-rich bromine atoms also interact with the A core at a 3.6 Å distance (Fig. 3c). These two factors are responsible for strong CT interaction, resulting in highly redshifted CT phosphorescence. In addition, the halogen–carbonyl interactions (3.0 Å and 3.8 Å) were also present in the segregated stacks, resulting in phosphorescence emission (Fig. 3c). Therefore, the presence of multifarious interactions engendered tunable phosphorescence with reasonable efficiency in all three cocrystals. Furthermore, we could achieve similar spectroscopic characteristics in spin-coated film states that extended the suitability of the current approach for different applications (Fig. 2c and S9, Table S8†).

## Conclusions

In this study, we have successfully achieved tunable charge-transfer phosphorescence across a broad range of 100 nm using three organic donor–acceptor cocrystals (A–D_1_, A–D_2_, and A–D_3_) through a simple yet effective cocrystal strategy. The combination of π–stacking interactions and multiple, directional halogen–carbonyl interactions synergistically resulted in the realization of room-temperature phosphorescence from the triplet CT states, exhibiting reasonable quantum yields in all three cocrystals. Notably, our approach involved an effortless synthesis and harnessed the power of non-covalent (supramolecular) through-space CT interactions, enabling the emission to span from cyan to green to yellow to orange regimes. The pronounced stoke-shifted emission, facilitated by the strong stabilization of CT interactions, presents promising opportunities in the fields of visible light catalysis and imaging. Additionally, the strong CT interaction played a crucial role in inducing a minor TADF contribution, which unlocks significant potential for advancing research and applications in these domains. Our study opens up a plethora of new possibilities for utilizing organic charge-transfer cocrystals in ambient triplet harvesting and designing organic phosphors, extending beyond their well-explored applications in organic electronics and ferroelectrics.^9^

## Data availability

All the experimental data were provided in the ESI.†

## Author contributions

AAK carried out all the experiments directed by SJG and analyzed the data. SNA solved the crystal structure. AAK and SMW synthesized the molecules. AAK, SG and SJG wrote the manuscript with contributions from all authors.

## Conflicts of interest

There are no conflicts to declare.

## Supplementary Material

SC-014-D3SC04001A-s001

SC-014-D3SC04001A-s002
