# Supplementary material for: Revisiting organic charge-transfer cocrystals for wide-range tunable, ambient phosphorescence
Source: Chem Sci. 2023 Oct 31;14(44):12548–53. doi: 10.1039/d3sc04001a (PMC10646860; doi:10.1039/d3sc04001a)
Supplement: SC-014-D3SC04001A-s001 [file SC-014-D3SC04001A-s001.pdf]

Electronic Supplementary Material (ESI) for Chemical Science.
This journal is © The Royal Society of Chemistry 2023

Supporting Information

Revisiting organic charge-transfer cocrystals for wide-range tunable, ambient phosphorescence

Anju Ajayan Kongasseri, Shagufi Naz Ansari, Swadhin Garain, Sopan M. Wagalgave and Subi J. George*

New Chemistry Unit and School of Advanced Materials, Jawaharlal Nehru Centre for Advanced Scientific Research (JNCASR) Jakkur, Bangalore 560064, India

Table of Contents

1. General Methods

2. Synthetic Schemes and Procedures

3. Experimental Procedures

4. Supporting Figures

5. Characterization Data

6. References

1. General Methods

NMR Measurements: ^{1}H and ^{13}C NMR spectra were recorded on a BRUKER AVANCE-400 fourier transformation spectrometer with 400 and 100 MHz respectively. The spectra were calibrated with respect to the residual solvent peaks. The chemical shifts are reported in parts per million (ppm) with respect to TMS. Short notations used are, s for singlet, d for doublet, t for triplet, q for quartet and m for multiplet.

Spectroscopic Measurements: Excitation and emission spectra were recorded on FLS1000 spectrometer, Edinburgh Instruments. Fluorescence spectra of the films were recorded in front-face geometry to avoid self-absorption.

Lifetime and quantum yield measurements: Phosphorescence lifetime, gated emission and time-resolved excitation and emission were measured on FLS1000 spectrometer, Edinburgh Instruments equipped with a micro flash-lamp (μF2) set-up. Fluorescence lifetimes were performed on the same instrument. A 405 nm laser diode with a pulse repetition rate of 20 MHz were used as the light sources. Quantum yields were measured using an integrating sphere in the same instrument.

UV-Vis Spectroscopy: UV-Vis absorption studies were performed in solid state using Agilent Cary-5000 UV-Vis-NIR spectrophotometer instrument.

High Resolution Mass Spectrometry (HR-MS): HR-MS was carried out using Agilent Technologies 6538 UHD Accurate-Mass Q-TOFLC/MS.

Single crystal X-ray crystallography: Suitable single crystal of **A-D$_1$, A-D$_2$** and **A-D$_3$** were mounted on a thin glass fibre with commercially available super glue. Intensity data were collected Bruker D8 VENTURE diffractometer equipped with a PHOTON detector and graphite-monochromated Mo-Kα radiation (λα = 0.71073 Å, 50 kV, 1mA) at 100 K. APEX III software was used to collect, reduce and integrate the raw data. The direct method was used for solving crystal structure, followed by full-matrix least-squares refinements against F2 (all data HKLF 4 format) using the SHELXL 2014/7 and difference Fourier synthesis and least-squares refinement revealed the positions of the non-hydrogen atoms. All nonhydrogen atoms were refined anisotropically and remaining hydrogen atoms were placed in geometrically constrained positions and refined with isotropic temperature factors, generally 1.2 × Ueq of their parent atoms. Molecular structure drawings were prepared using the program Mercury (ver 3.1).

2. Synthetic Scheme and Procedure

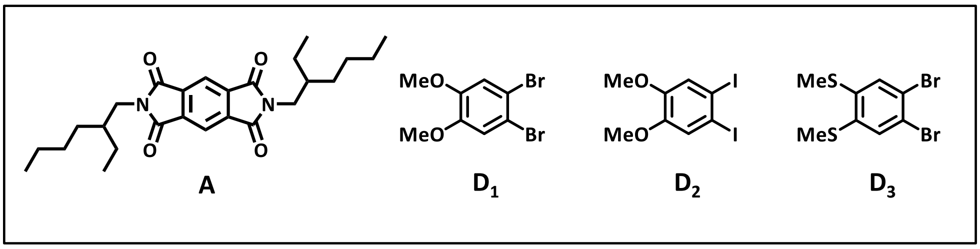

Scheme S1. Molecular Structures of **A**, **D_1**, **D_2** and **D_3**.

Pyromellitic diimide (**A**) was synthesized according to the literature procedure. [S1]

The donors **D_1**, **D_2** and **D_3** were synthesized as follows.

1,2-dimethoxybenzene, 1,2-dithiophenol, periodic acid, bromine, iodomethane, iodine and solvents were purchased from Sigma Aldrich, TCI and spectrochem and used without further purification.

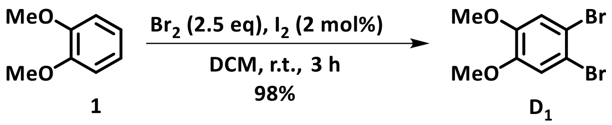

Scheme S2. Synthetic scheme for **D_2**.

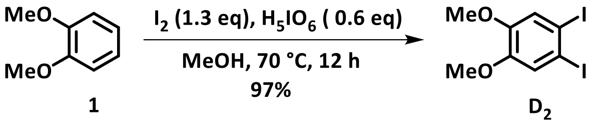

Scheme S3. Synthetic scheme for **D_2**.

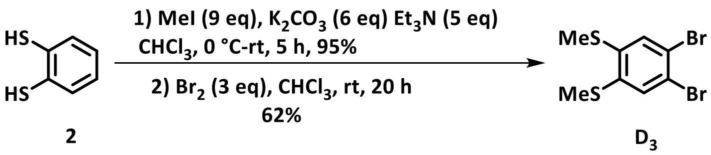

Scheme S4. Synthetic scheme for **D_3**.

Synthesis of 1,2 dibromo-4,5-dimethoxybenzene (D_1): 1,2-Dimethoxybenzene (2 g, 14.47 mmol) and iodine (73 mg, 0.29 mmol) were dissolved in dichloromethane (40 mL). Bromine (5.78 g, 36.19 mmol) was added dropwise for 10 min via a dropping funnel to the reaction mixture. The reaction mixture colour was changed from purple to yellow-brown, and after the addition of Br_2 the mixture was stirred at room temperature for 3 h. The reaction mixture was concentrated up to dryness on vacuum. The crude product was dissolved in hot isopropanol (40 mL). The solution was cooled (-20 °C) for 24 h. The resulting **D_1** was isolated by filtration and dried under vacuum to produce colourless needles (4.20 g, 98% yield). ^{1}H NMR (400 MHz, $CDCl_3$) δ (ppm) 7.06 (s, 2H), 3.86 (s, 6H); ^{13}C NMR (100 MHz, $CDCl_3$) δ

(ppm) 148.93, 115.98, 114.80, 56.30; HRMS (APCI): m/z calculated for $C_8H_8Br_2O_2$: 295.8871; observed 295.8847 $[M]^+$.

Synthesis of 1,2 diiodo-4,5-dimethoxybenzene (D_2): Periodic acid (2.11 g, 9.26 mmol) was dissolved in methanol (20 mL). Iodine (4.77 g, 18.82 mmol) was added, and the mixture obtained was stirred for 20 min under room temperature. 1,2-dimethoxybenzene (2 g, 14.45 mmol) was added and the mixture was refluxed at 70 °C for 4 h. The reaction mixture was allowed to cool at room temperature and poured into a solution of sodium sulfite (10 g in 100 mL water). The white precipitate was isolated by filtration, washed with cold methanol (20 mL) and air-dried. To obtain the desired white solid **D_2** (5.48 g, 97% yield). ^{1}H NMR (400 MHz, $CDCl_3$) δ (ppm) 7.24 (s, 2H), 3.84 (s, 6H); ^{13}C NMR (100 MHz, $CDCl_3$) δ (ppm) 149.65, 121.72, 96.06, 56.18; HRMS (APCI): m/z calculated for $C_8H_8I_2O_2$: 389.8614; observed 389.8583 $[M]^+$.

Synthesis of 1,2 diiodo-4,5-bis(methylsulfanyl)benzene (D_3): 1,2-dithiophenol (200 mg, 1.40 mmol) and K_2CO_3 (1.16 g, 8.45 mmol) were dissolved in chloroform (10 mL). The reaction mixture was cool to 0 °C and Et_3N (980 µL, 7.04 mmol) and iodomethane (789 µL, 12.67 mmol) were added. The reaction mixture was stirred at room temperature for 5 h and the reaction was monitored by TLC. HCl (2 M, 5 mL) was added, and the mixture was extracted by dichloromethane (10 mL×3). The combined organic layers were washed with brine (20 mL), dried over sodium sulfate, filtered, and concentrated under a vacuum. The residue was purified by silica gel column chromatography (hexane/AcOEt) to afford 1,2-bis(methylsulfanyl)benzene (228 mg, 95%) as a yellow oil. To the suspension of 1,2-bis(methylsulfanyl)benzene (200 mg, 1.17 mmol) dissolved in $CHCl_3$ (15 mL), bromine (563 mg, 3.52 mmol) was added. The reaction mixture was stirred at room temperature for 20 h. The excess of bromine was removed by sodium sulfite, and the product was purified by column chromatography over silica gel (Hexane/AcOEt) to obtain the desired white solid **D_3** (240 mg, 62% yield). ^{1}H NMR (400 MHz, $CDCl_3$) δ (ppm) 7.35 (s, 2H), 2.47 (s, 6H); ^{13}C NMR (100 MHz, $CDCl_3$) δ (ppm) 138.46, 138.44, 130.42, 130.36, 121.54, 121.53, 16.33; HRMS (APCI): m/z calculated for $C_8H_8Br_2S_2$: 327.8414; observed 327.8395 $[M]^+$.

3. Experimental Procedures

Protocol for cocrystal synthesis: Saturated solutions of both donor and acceptors in chloroform were prepared and mixed in 1:1 molar ratio. Then ethanol was added into it as a bad solvent. The mixture was heated at 70 °C for ten minutes and then kept at room temperature for crystallization.

Protocol for sample preparation: For thin films, of D-A complexes, the saturated solutions of D and A was drop casted on to a clean quartz substrate and kept for drying. For preparing 1 wt% thin films, acceptor or donor molecules (1 mg) were mixed with 100 mg of PMMA. This mixture was then heated at 50 °C for 10 minutes followed by sonication (5 minutes) to dissolve all the components thoroughly. Then, 0.5 mL of this solution was drop-casted on a clean quartz substrate. Finally, the drop-casted thin films were dried at 60 °C for 30 minutes before performing the photophysical studies. For the phosphorescence studies of the cocrystals, a small amount of the cocrystal was placed in between two quartz plates and is properly sealed.

4. Supporting Figures

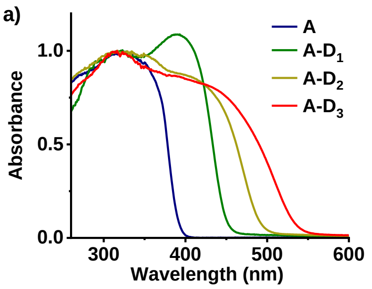

Figure S1: UV-Vis absorption spectra of **A**, **A-D₁**, **A-D₂**, and **A-D₃** cocrystals.

Note: The red-shifted absorption band of cocrystals, when compared to acceptor alone, suggested the ground-state charge-transfer complexation in the cocrystals.

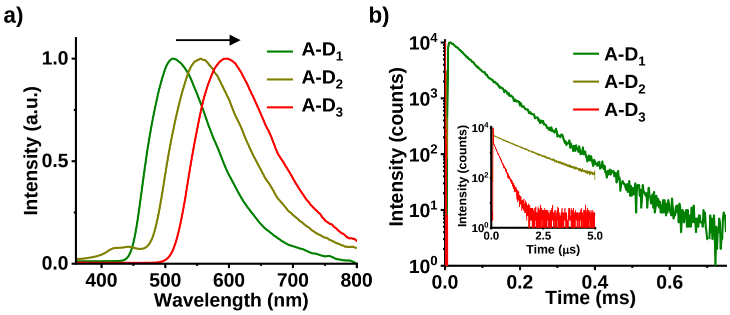

Fig. S2. a) Emission spectra of **A-D₁**, **A-D₂** and **A-D₃** cocrystals ($\lambda_{exc.}$ = 340 nm). b) Lifetime decay plots measured upon exciting at CT band of **A-D₁** ($\lambda_{exc.}$ = 430 nm, $\lambda_{collected}$=515 nm τ = 95.10 µs, green line)**,** **A-D₂** ($\lambda_{exc.}$ = 405 nm, $\lambda_{collected}$=555 nm τ = 1.51 µs, yellow line), and **A-D₃** ($\lambda_{exc.}$ = 405 nm, $\lambda_{collected}$=595 nm τ = 0.22 µs, red line).

Note: Emission profile upon exciting at locally-excited band is same as that when excited at the charge-transfer band for all the cocrystals (Fig. 2a).

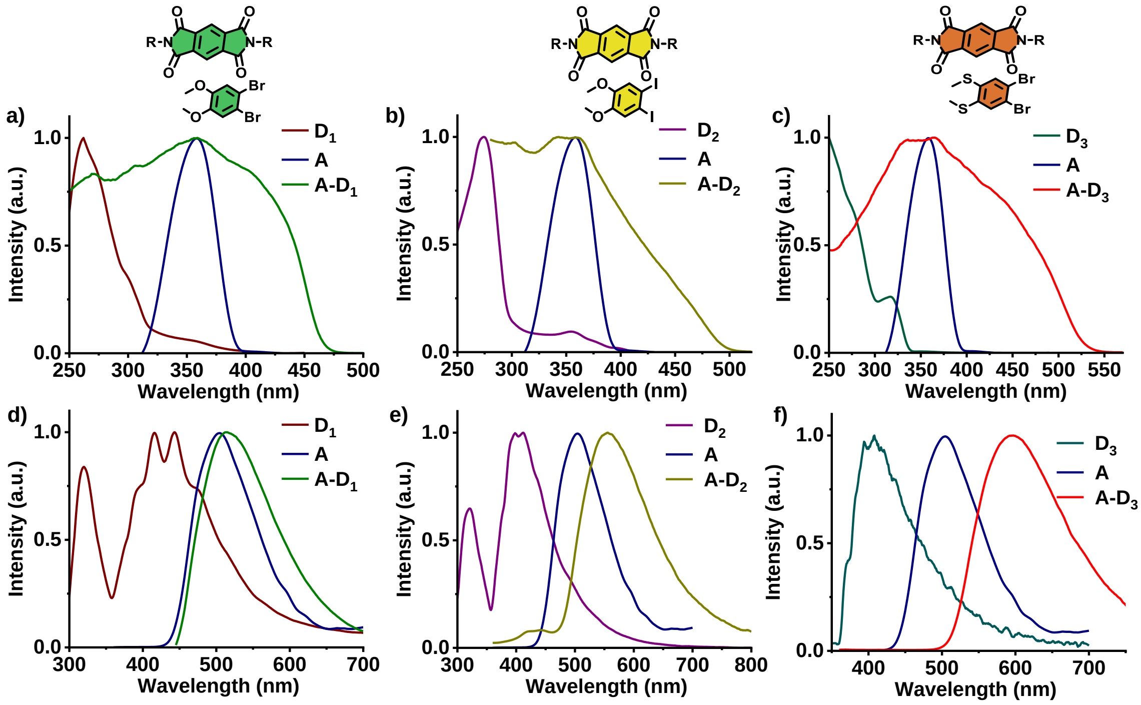

Figure S3. Comparison of the photophysical properties of individual donors and acceptor with cocrystals. a) Normalized excitation spectra of $\mathbf{D_1}$ (maroon line) ($\lambda_{monitored}$ = 430 nm), **A** (navy blue line) ($\lambda_{monitored}$ = 500 nm) and $\mathbf{A\text{-}D_1}$ (green line) ($\lambda_{monitored}$ = 515 nm), b) Normalized excitation spectra of $\mathbf{D_2}$ (purple line) ($\lambda_{monitored}$ = 430 nm), **A** (navy blue line) ($\lambda_{monitored}$ = 500 nm) and $\mathbf{A\text{-}D_2}$ (dark yellow line) ($\lambda_{monitored}$ = 555 nm), c) Normalized excitation spectra of $\mathbf{D_3}$ (bluish green line) ($\lambda_{monitored}$ = 450 nm), **A** (navy blue line) ($\lambda_{monitored}$ = 500 nm) and $\mathbf{A\text{-}D_3}$ (red line) ($\lambda_{monitored}$ = 595 nm), d) Normalized gated emission spectra of $\mathbf{D_1}$ (maroon line) ($\lambda_{exc.}$ = 275 nm, delay time = 1.00 ms), **A** (navy blue line) ($\lambda_{exc.}$ = 340 nm, delay time = 1.00 ms) and $\mathbf{A\text{-}D_1}$ (green line) ($\lambda_{exc.}$ = 430 nm, delay time = 50 µs), e) Normalized gated emission spectra of $\mathbf{D_2}$ (purple line) ($\lambda_{exc.}$ = 275 nm, delay time = 1.00 ms), **A** (navy blue line) ($\lambda_{exc.}$ = 340 nm, delay time = 1.00 ms)and steady-state emission spectrum of $\mathbf{A\text{-}D_2}$ (dark yellow line) ($\lambda_{exc.}$ = 450 nm) and f) Normalized gated emission spectra of $\mathbf{D_3}$ (bluish green line) ($\lambda_{exc.}$ = 275 nm, delay time = 1.00 ms), **PmDI** (navy blue line) ($\lambda_{exc.}$ = 340 nm, delay time = 1.00 ms) and and steady-state emission spectrum of $\mathbf{A\text{-}D_3}$ (red line) ($\lambda_{exc.}$ = 480 nm). The individual donors ($\mathbf{D_1}$, $\mathbf{D_2}$ and $\mathbf{D_3}$) and acceptor, **A** were doped in PMMA matrix, where 1 wt% of the molecule was doped in PMMA and measurements were carried out in cryogenic conditions (20 K), whereas the gated and steady-state spectra of cocrystals were performed under ambient conditions.

Note: The red-shifted emission of cocrystals with respect to individual donors and acceptor suggested the different origin of emission of the crystals, i.e., from the newly formed CT states in the cocrystal, and not the individual LE states.

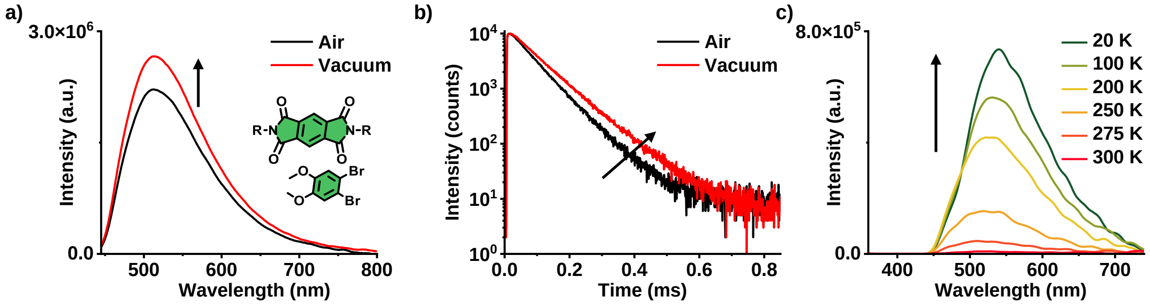

Fig. S4. Air and vacuum studies of **A-D₁**. a) Emission spectra and b) lifetime decay plots of **A-D₁** cocrystals. c) Emission spectra of **A-D₁** cocrystals at varying temperatures ($\lambda_{exc.}$ = 430 nm, $\lambda_{collected}$ = 515 nm for all measurements).

Note: The increase in the emission intensity and lifetime under vacuum compared to that of air suggested the involvement of triplet states. The increasing emission intensity with decreasing temperature confirmed the nature of emission to be phosphorescence.

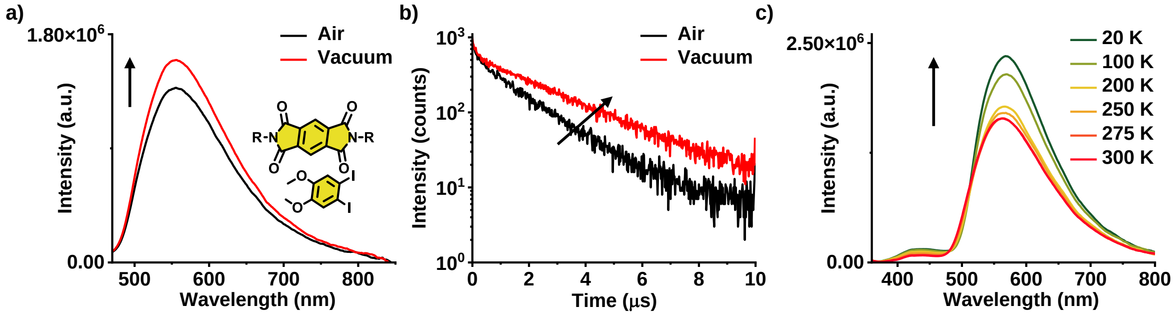

Fig. S5. Air and vacuum studies of **A-D₂**. a) Emission spectra and b) lifetime decay plots. c) Emission spectra of **A-D₂** cocrystals at varying temperatures. ($\lambda_{exc.}$ = 450 nm for emission studies, $\lambda_{exc.}$ = 405 nm, $\lambda_{collected}$ = 555 nm for lifetime decay studies)

Note: The increase in the emission intensity and lifetime under vacuum compared to that of air suggested the involvement of triplet states. The increasing emission intensity with decreasing temperature confirmed the nature of emission to be phosphorescence.

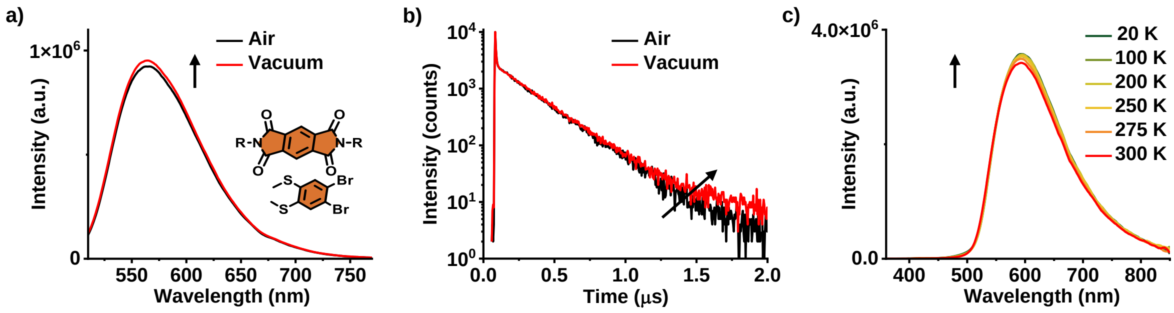

Fig. S6. Air and vacuum studies of **A-D₃** . a) Emission spectra and b) lifetime decay plot of **A-D₃** cocrystals. c) Emission spectra of **A-D₃** cocrystals at varying temperatures ($\lambda_{exc.}$ = 480 nm for emission studies and, $\lambda_{exc.}$ = 405 nm, $\lambda_{collected}$ = 595 nm for lifetime decay measurements).

Note: The increase in the emission intensity and lifetime under vacuum compared to that of air suggested the involvement of triplet states. The shorter component of lifetime increased and the

longer component decreased on increasing temperature, suggesting presence of both TADF and phosphorescence simultaneously.

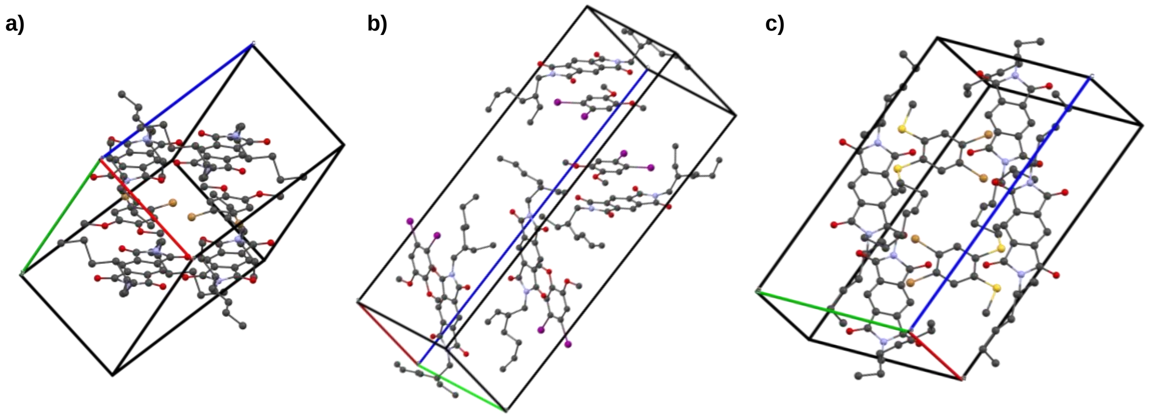

Fig. S7. Unit cells of a) **A-D$_1$**, b) **A-D$_2$** and c) **A-D$_3$**. CCDC numbers are 2201065, 2197363 and 2196177, respectively.

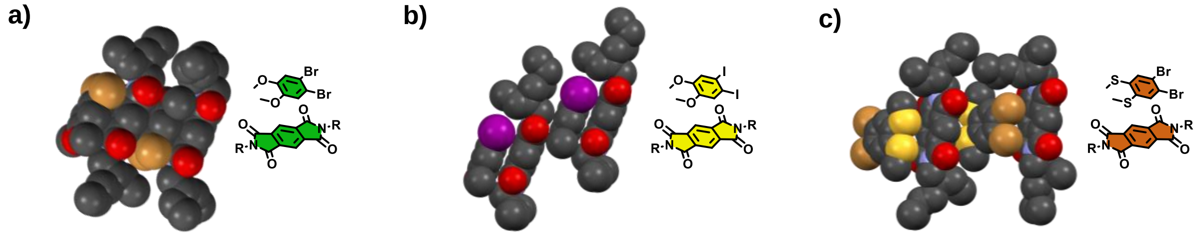

Fig. S8. Single-crystal X-ray diffraction data showing the π-π stacking of cocrystal structures shown in spacefill form a) A-D$_1$, b) A-D$_2$ and c) A-D$_3$ cocrystals.

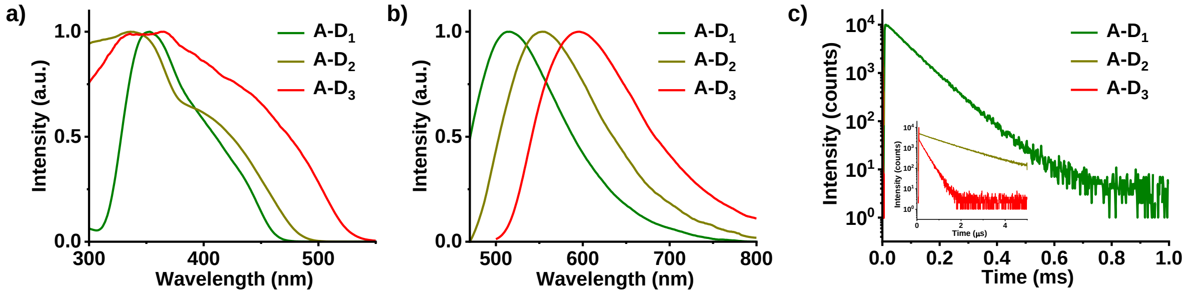

Fig. S9. a**)** Normalized excitation spectra of D-A complexes in film state **A-D$_1$ (**$\lambda_{monitored}$ = 515 nm, green line), **A-D$_2$** ($\lambda_{monitored}$ = 555 nm, yellow line) and **A-D$_3$** ($\lambda_{monitored}$ = 595 nm, red line). b) Steady-state emission spectra of **A-D$_1$** ($\lambda_{exc.}$ = 430 nm), **A-D$_2$** ($\lambda_{exc.}$ = 450 nm) and **A-D$_3$** ($\lambda_{exc.}$ = 480 nm), showing gradual red-shift in the emission maxima while progressing from **A-D$_1$**, **A-D$_2$** and **A-D$_3$**. c) Lifetime decay plots measured upon exciting at CT band of **A-D$_1$** ($\lambda_{exc.}$ = 430 nm, $\lambda_{collected}$=515 nm, green line), **A-D$_2$** ($\lambda_{exc.}$ = 405 nm, $\lambda_{collected}$ = 555 nm, yellow line), and **A-D$_3$** ($\lambda_{exc.}$ = 405 nm, $\lambda_{collected}$= 595 nm, red line).

Note: Spectroscopic characteristics in film state is similar to that of the cocrystals.

Table S1. Summary of lifetime decay profiles of **A-D$_1$**, **A-D$_2$** and **A-D$_3$** cocrystals under ambient conditions.

	**$\lambda_{exc.}$ (nm)**	**$\lambda_{monitored}$ (nm)**	τ_1	τ_2	**$<\tau_{avg.}>$**
**A-D$_1$**	430	515	79.41 µs (53 %)	112.80 µs (47 %)	95.10 µs
**A-D$_2$**	405	555	0.20 µs (6 %)	1.59 µs (94 %)	1.51 µs
**A-D$_3$**	405	595	4.55 ns (5 %)	0.23 µs (95 %)	0.22 µs

Table S2. Summary of lifetime decay profiles of **A-D$_1$** cocrystals in air and vacuum.

	**$\lambda_{exc.}$ (nm)**	**$\lambda_{monitored}$ (nm)**	**τ_1 (µs)**	**τ_2 (µs)**	**$<\tau_{avg.}>$ (µs)**
**Air**	430	515	68.52 (42 %)	108.07 (58 %)	91.46
**Vacuum**	430	515	79.08 (51 %)	115.60 (49 %)	96.97

Table S3. Summary of lifetime decay profiles of **A-D$_2$** cocrystals in air and vacuum.

	**$\lambda_{exc.}$ (nm)**	**$\lambda_{monitored}$ (nm)**	**τ_1 (µs)**	**τ_2 (µs)**	**$<\tau_{avg.}>$ (µs)**
**Air**	405	555	0.21 (4 %)	1.62 (96 %)	1.56
**Vacuum**	405	555	0.19 (3 %)	2.61 (97 %)	2.54

Table S4. Summary of lifetime decay profiles of **A-D$_3$** cocrystals in air and vacuum.

	**$\lambda_{exc.}$ (nm)**	**$\lambda_{monitored}$ (nm)**	**τ_{1i} (µs)**	**τ_2 (µs)**	**$<\tau_{avg.}>$ (µs)**
**Air**	405	595	5.03 (7 %)	0.23 (93 %)	0.21
**Vacuum**	405	595	5.75 (5 %)	0.24 (95%)	0.23

Table S5. Summary of lifetime decay profiles of **A-D$_1$** cocrystals at varying temperatures.

Temperature (K)	$\lambda_{exc.}$ (nm)	$\lambda_{monitored}$ (nm)	τ_1 (µs)	τ_2 (µs)	$<\tau_{avg.}>$ (µs)
**20**	430	515	353.60 (34 %)	647.30 (66 %)	547.44
**300**	430	515	88.61 (60 %)	125.30 (40 %)	103.29

Table S6. Summary of lifetime decay profiles of **A-D$_2$** cocrystals at varying temperatures.

Temperature (K)	$\lambda_{exc.}$ (nm)	$\lambda_{monitored}$ (nm)	τ_1 (µs)	τ_2 (µs)	$<\tau_{avg.}>$ (µs)
**20**	405	555	0.68 (4%)	34.58 (96 %)	33.22
**300**	405	555	0.30 (9 %)	2.76 (91 %)	2.54

Table S7. Summary of lifetime decay profiles of **A-D$_3$** cocrystals at varying temperatures.

Temperature (K)	$\lambda_{exc.}$ (nm)	$\lambda_{monitored}$ (nm)	τ_1 (ns)	τ_2 (µs)	$<\tau_{avg.}>$ (µs)
**20**	405	595	8.26 (11 %)	0.32 (89 %)	0.29
**300**	405	595	5.26 (6 %)	0.25 (94 %)	0.24

Table S8. Summary of lifetime decay profiles of **A-D$_1$**, **A-D$_2$** and **A-D$_3$** CT complexes in film state.

	$\lambda_{exc.}$ (nm)	$\lambda_{monitored}$ (nm)	τ_1	τ_2	$<\tau_{avg.}>$
**A-D$_1$**	430	515	58.13 µs (49 %)	98.18 µs (51 %)	78.55 µs
**A-D$_2$**	405	555	0.15 µs (2 %)	1.22 µs (98 %)	1.19 µs
**A-D$_3$**	4050	595	5.21 ns (7 %)	0.19 µs (93 %)	0.18 µs

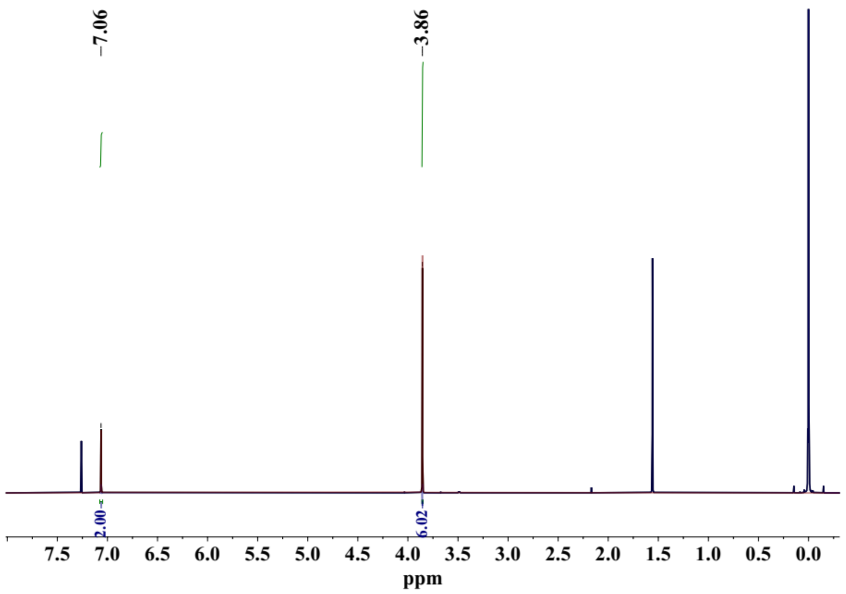

Fig. S10. ^{1}H NMR spectrum of $\mathbf{D_1}$ in $CDCl_3$.

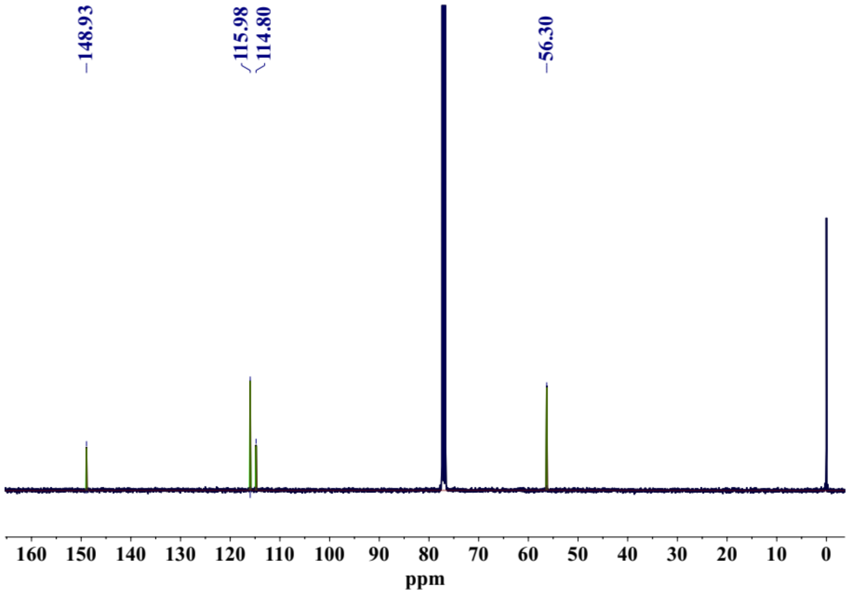

Fig. S11. ^{13}C NMR spectrum of $\mathbf{D_1}$ in $CDCl_3$.

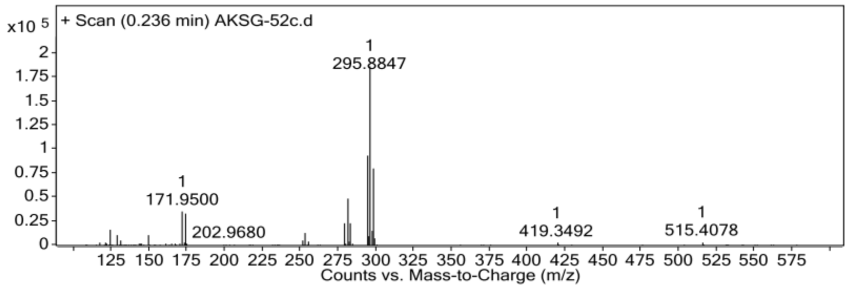

Fig. S12. APCI-HR-MS spectrum of $\mathbf{D_1}$.

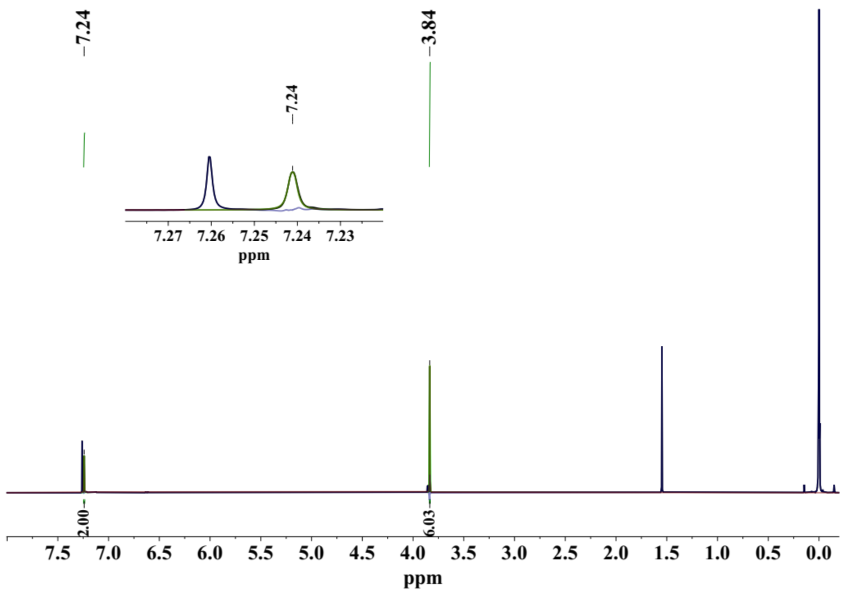

Fig. S13. ^{1}H NMR spectrum of $\mathbf{D_2}$ in $CDCl_3$.

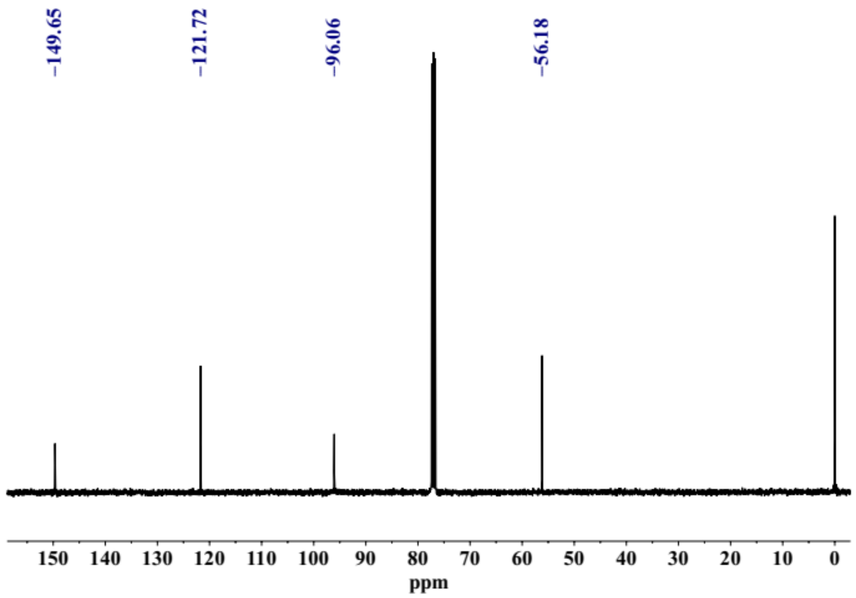

Fig. S14. ^{13}C NMR spectrum of $\mathbf{D_2}$ in $CDCl_3$.

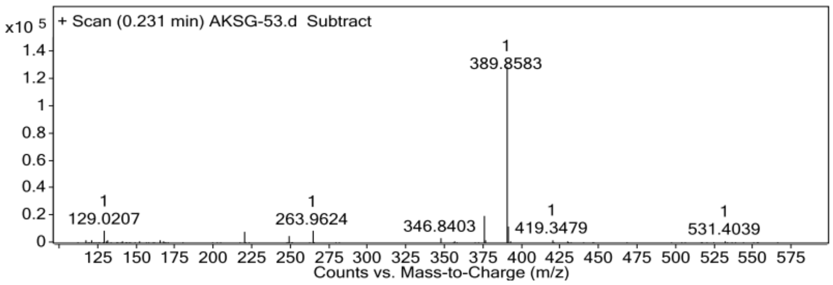

Fig. S15. APCI-HR-MS spectrum of $\mathbf{D_2}$.

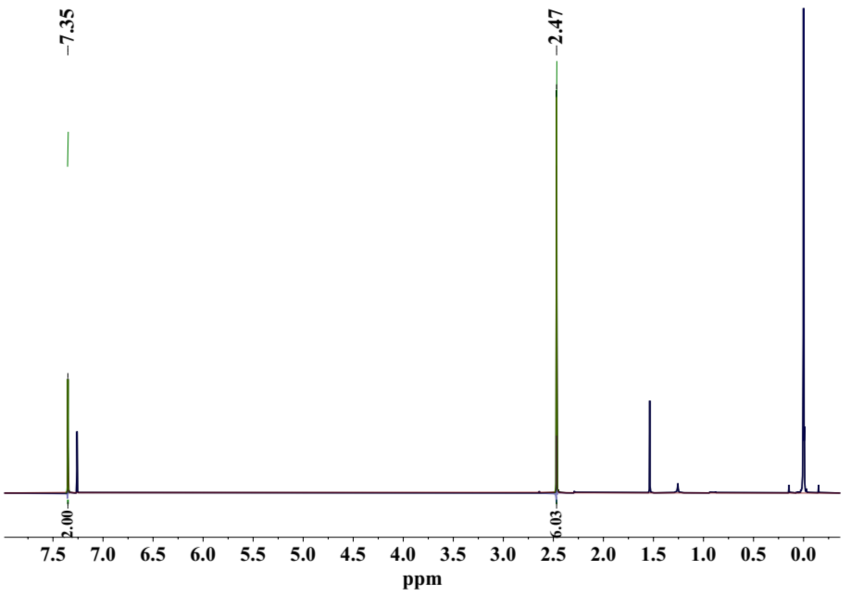

Fig. S16. ^{1}H NMR spectrum of **D$_3$** in $CDCl_3$.

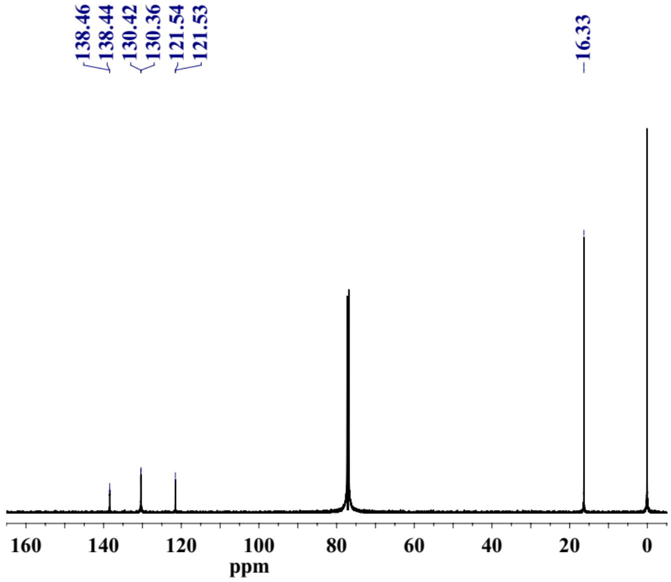

Fig. S17. ^{13}C NMR spectrum of **D$_3$** in $CDCl_3$.

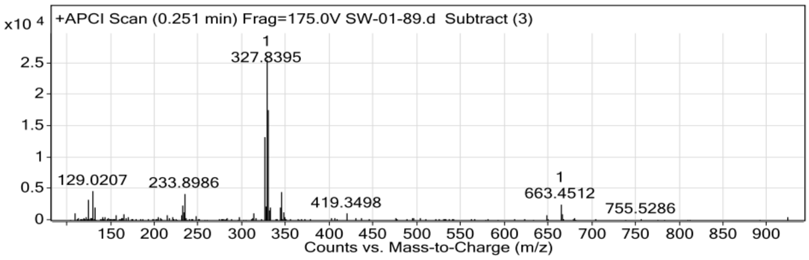

Fig. S18. APCI-HR-MS spectrum of **D$_3$**.

6. References

[1] S. Garain, S. N. Ansari, A. A. Kongasseri, B. C. Garain, S. K. Pati and S. J. George, *Chem. Sci.,* 2022, **13**, 10011–10019.
